# Gene Therapy for Spinal Muscular Atrophy (SMA): A Review of Current Challenges and Safety Considerations for Onasemnogene Abeparvovec (Zolgensma)

**DOI:** 10.7759/cureus.36197

**Published:** 2023-03-15

**Authors:** Tolu Ogbonmide, Rajni Rathore, Shahid B Rangrej, Stedrea Hutchinson, Marcia Lewis, Stephenie Ojilere, Victoria Carvalho, Irenaissia Kelly

**Affiliations:** 1 College of Medicine, Saint James School of Medicine, Saint Vincent and Grenadines, VCT; 2 Pharmacology and Therapeutics, Saint James School of Medicine, Saint Vincent and grenadines, VCT; 3 Anatomy/Research, Saint James School of Medicine, Saint Vincent and Grenadines, VCT; 4 Pharmacology, Saint James School of Medicine, Saint Vincent and Grenadines, VCT; 5 Neurology, Saint James School of Medicine, Saint Vincent and Grenadines, VCT

**Keywords:** gene therapy, avxs-101, spinal muscular atrophy, spinal muscular atrophy treatment for infant, gene-replacement therapy, sma 2, sma 1, sma, onasemnogene abeparvovec, zolgensma

## Abstract

Spinal Muscular Atrophy (SMA) is a genetic disease that causes weakness and wasting in the voluntary muscles of infants and children. SMA has been the leading inherited cause of infant death. More specifically, SMA is caused by the absence of the SMN1 gene. In May 2019, the Food and Drug Administration (FDA) approved onasemnogene abeparvovec, SMN1 gene replacement therapy, for all children with SMA younger than two years of age, without end-stage weakness. The objective of the study is to review the safety and efficacy of a novel gene therapy, onasemnogene abeparvovec (Zolgensma), for SMA and assess current challenges for gene therapy. For this, we have conducted a literature search on PubMed, MEDLINE, and Ovid (2019 to 2022) in the English language using the terms SMA, onasemnogene, and gene therapy. The search included articles, websites, and published papers from reputable health organizations, hospitals, and global organizations dedicated to bringing awareness to Spinal Muscular Atrophy. We found the first gene therapy for SMA to be onasemnogene, directly providing the survival motor neuron 1 (SMN1) gene to produce the survival motor neuron (SMN) protein. Onasemnogene is approved by the Food and Drug Administration and has the added benefit of being a one-time dose. On the downside, a major side effect of this treatment is hepatotoxicity. There is substantial evidence that the efficacy of therapy is increased when administered early to children under three months of age. Therefore, we concluded that onasemnogene appears to be an efficacious therapy for younger pediatric patients with SMA type 1. Drug cost and potential hepatotoxicity are major concerns. Long-term benefits and risks have not been determined, but it is more cost-effective and requires less time of treatment compared to the other used drug, nusinersen. Therefore, the combined safety, cost, and effectiveness of onasemnogene abeparvovec make it a reliable treatment option for treating SMA Type 1.

## Introduction and background

SMA is a genetic disease that causes weakness and wasting in the voluntary muscles of infants and children and, more rarely, in adults [1]. It is identified as the loss of lower motor neurons in both the spinal cord and brainstem, leading to progressive symmetrical muscle weakness [2]. Specifically, it is an autosomal recessive disorder in the survival motor neuron 1 gene, SMN1, that causes a loss of specialized nerve cells, termed alpha motor neurons that control muscle movement. The SMN protein is vital for its role in the spliceosome assembly and biogenesis of ribonucleoproteins. Recent studies have demonstrated that the SMN protein role ranges from other cell processes such as mRNA trafficking, local translation, cytoskeletal dynamics, endocytosis, and autophagy. Therefore, a loss of the SMN protein affects the motor neuron’s homeostatic environment [3]. A loss of these motor neurons prevents the sending of signals between the spinal cord and skeletal muscle, resulting in progressive proximal muscle weakness and paralysis [4].

The SMA phenotype is categorized into four grades of severity (SMA I, SMA II, SMA III, SMA IV) based on the age of onset and motor function achieved [5]. Type 1 is the most severe, where the patient is unable to sit; Type 2 is unable to walk unaided; Type 3 is able to achieve some walking abilities; and Type 4 is adult-onset SMA [6,7]. SMN2 copy amount largely accounts for the clinical severity between the SMA types, with other genetic or environmental factors playing only a minor role. The higher the amount of SMN2 copy mutation, the higher the probability of developing a severe phenotype. Hence, the higher the amount of SMN2 types, the greater the clinical severity. When untreated, it will result in severe limitations to motor function, including walking incapability; high risk for respiratory complications resulting in the need for some degree of ventilatory support; as well as a high risk for orthopedic complications such as frequently painful contractures and scoliosis; and reduced life expectancy. 45% to 60% of cases of SMA are SMA Type 1, making it the most common form of SMA [8]. Patients with SMA Type 1 with 2 copies of SMN2 have a particularly poor prognosis. These patients usually show signs of SMA before six months of age, evident by their lack of ability to sit. Unfortunately, these infants typically do not survive past two years of age without significant mechanical ventilatory and nutritional support [8]. The SMN2 gene is a highly homologous copy of the SMN1 gene and hence, is considered a phenotypic modifier of the SMA disease. The greater the number of copies of the SMN2 genes, the less severe the clinical presentation of SMA [6]. The incidence of SMA is often cited as being approximately 10 in every 100,000 live births, whereas the prevalence is estimated at approximately 1 to 2 in every 100,000 people [9]. The diagnosis of SMA can be confirmed with molecular genetic testing with targeted mutation analysis [10] because mutations in the SMN1 gene cause spinal muscular atrophy (SMA), a disorder characterized by progressive symmetric muscle weakness that can be complicated by other features, including joint contractures, scoliosis, growth failure, and restrictive lung disease. Mutation testing is done by selecting a set of mutation operators and then applying them to the source program one at a time for each applicable piece of the source code.

Gene therapy is an experimental approach that uses imported genes to treat disorders that result from genetic mutations [11]. Gene therapies include replacing, silencing, or knocking out a mutated gene or introducing a new gene to restore additional function or protection [9]. Onasemnogene abeparvovec, also known as Zolgensma, is a gene therapy that was recently approved by the US Food and Drug Administration as a treatment for SMA in pediatric patients under the age of two in May 2019. Zolgensma consists of a single-dose, free-of-preservative, sterile, intravenous infusion of a non-replicating, self-complementary adeno-associated vector 9 (AAV9) that crosses the blood-brain barrier. The active substance in Zolgensma contains a functional copy of the SMN1 gene under the control of the cytomegalovirus (CMV) enhancer/chicken-β-actin-hybrid promoter (CB). One of the two adeno-associated vectors (AAV) inverted terminal repeats (ITRs) has been modified to promote intramolecular annealing of the transgene, thus forming a double-stranded transgene ready for transcription. Therefore, restoring a normal SMN protein, which regulates cellular homeostatic pathways and, by extension, the state of the motor neuron [8].

In 2018, SMA was added to the Recommended Uniform Screening Panel for Newborns in the United States, and several states have since adopted SMA newborn screening [12]. With diagnoses being made through screening practices, treatments are to be considered. Comparisons between the US Spinal Muscular Atrophy standard mode of care, nusinersen, and the promising alternative Zolgensma are presented to patients [13]. This review investigates the effectiveness, safety concerns, and challenges in the distribution and accessibility of Zolgensma.

## Review

Methodology

Literature Search

Our review commenced with a literature search in Jan 2021, using electronic databases DynaMed and Access Medicine as well as PubMed, Embase, and ClinicalTrial.gov. To find articles with information relevant to our research question, we incorporated search words such as “onasemnogene abeparvovec”, “spinal muscular atrophy”, “AVXS101”, “safety”, “challenges” and “gene therapy” with the use of the Boolean operator “AND”. The inclusion criteria for articles selected were: (1) must be from a credible source; (2) published within the last five years; and (3) written in the English language.

Selection of Resources

The resources selected for inclusion in this narrative review provide pertinent information about Zolgensma as a gene therapy for SMA Type 1. These resources evaluate the cost, efficacy, safety, community priority of distribution, challenges, and accessibility of Zolgensma as a treatment option for SMA in children under the age of 2 years. The search produced 45 reputable sources. The studies performed (e.g., randomized and non-randomized controlled trials [RCTs]), review articles were included, and case-control, cohort (prospective/retrospective), and cross-section studies were excluded. No duplicates were contributed. To highlight the effect of Zolgensma on patients with SMA Type 1, 5 studies were included in a table providing a visual profile of the gene therapy. The Children’s Hospital of Philadelphia Infant Test of Neuromuscular Disorders (CHOP INTEND) is a scale used in patients diagnosed with pediatric spinal muscular atrophy to determine strength and range of motion in the patient. This is significant because it provides a consistent scale used by physicians all over the world to determine the overall muscle function of a child with SMA and its progression before and after treatment. Each criterion has a score ranging from 0 to 4 points, with the highest score being 64 points. Patients with SMA typically have a lower score than those not diagnosed. 

Results

Our study reviewed 45 articles from various sources, including PubMed, the National Institute for Clinical Excellence, and the U.S. National Library of Medicine. Out of the 45 articles, 10 were found to meet the above inclusion criteria. Of the 10 studies, five were selected to accurately portray the effects of Zolgensma on children under two years of age.

In one study [14], health outcomes in SMA Type 1 following Zolgensma gene replacement therapy were examined. The study included 12 participants. Of the total participants, seven infants did not require noninvasive ventilation at the end of the study. Eleven patients had stable or improved swallowing function and were also able to speak. Eleven patients achieved full head control and sat unassisted, and two patients were walking independently. All 15 patients were 20 months of age or younger.

After the Single-Dose Gene-Replacement Therapy for Spinal Muscular Atrophy study [15], all 15 patients survived treatment and improved over time. All patients were two years old or younger. After gene delivery in the high-dose cohort, according to the CHOP INTEND scale, patients experienced a rapid increase from baseline in the score. More specifically, at one month, an increase of 9.8 points was observed, and at three months, a 15.4-point increase. Out of the 15, 11 were able to sit without assistance, 11 could be orally fed and speak, nine were able to roll over, and two could walk independently.

The 2019 study [16-18] by Kevin Strauss et al. produced promising results. It was noted that none of the 15 participants used ventilatory or feeding tube support at any time. Eight of the 15 participants in this cohort study showed notable improvement. Two patients achieved the primary efficacy endpoint of standing alone within the typical developmental window of the same age range. Six other patients also walked alone within the typical developmental window of the same age range. All patients were 20 months of age or younger.

In the symptomatic SMA studies of Zolgensma, the START study [17] enrolled and divided 15 SMA patients with SMA Type 1 into two groups. The average age for Group 1 was 6.3 months, and 3.4 months for Group 2. Group 1 had 3 patients, ranging in age from 5.9 to 7.2 months, who were given a low dose of Zolgensma to make sure the drug was safe. The remaining 12 patients in Group 2, ranging in age from 0.9 to 7.9 months, were given a higher dose. The recommended dose of Zolgensma is 1.1 × 1014 vector genomes per kilogram (vg/kg) of body weight. Zolgensma is a suspension administered as a single intravenous infusion over 60 minutes. Zolgensma is provided as a kit customized to meet the weight-based dosing requirements of each patient. All vials have a nominal concentration of 2.0 × 1013 vector genomes (vg) per mL. Each vial of Zolgensma contains an extractable volume of not less than either 5.5 mL or 8.3 mL. Dose-volume for pediatric patients less than two years of age weighing equal to or greater than 13.6 kg will require a combination of Zolgensma kits. All patients were monitored up to two years of age. In group 2, all patients did not need permanent breathing support; 11 of them could sit for 5 seconds without support; 11 of 12 patients had a CHOP INTEND score greater than 40 points; and nine of the 12 could sit for 30 seconds without support. This study is now in its next phase, and more results are expected.

A study [5] on assessing the impact of age and motor function on SMA Type 1 infants who received a single dose of gene replacement therapy (The recommended dose of Zolgensma is 1.1 × 1014 vector genomes per kilogram (vg/kg) of body weight) demonstrated that the early dosing and low motor group, with a mean age of 17 months, had a mean gain of 35.0 points from a mean baseline (15.7). The late dosing group, on the other hand, with a mean age of 22.0 months, had a mean gain of 23.3 from a mean baseline of 26.5. The group of early-dosing and high-motor children, with a mean age of 9.4 months, reached a mean score of 60.3 out of a scale maximum of 64 and a baseline of 44.0. Even though the early dosing and low motor group had a lower baseline motor score, this group was able to sit unassisted earlier than the late dosing group. 

The National Institute for Health and Care Excellence addressed a setback in treating SMA with Zolgensma, namely that disadvantaged groups are not able to screen for the disease early enough for the treatment to be effective [19]. Hepatotoxicity is another disadvantage of Zolgensma because it can trigger an immune response that increases enzyme production in the liver [4]. The most commonly observed adverse reactions (incidence ≥5%) in clinical studies were elevated aminotransferases and vomiting [13].

Discussion

Zolgensma is a breakthrough therapy for SMA due to the one-time administration regimen associated with this medication. The one-time administration is in stark contrast to the treatment regimens of comparable therapies such as Evrysdi (risdiplam) and Spinraza (nusinersen). Evrysdi is a daily oral medication that must be taken for the duration of the individual’s life [20]. Spinraza is administered via an intrathecal injection with four loading doses in the first two months of treatment, followed by a maintenance dose every four months for the duration of the individual’s life [20]. Common treatment-related side effects include infusion site reactions, thrombocytopenia, renal toxicity, and liver function [21]. FDA approval of Zolgensma in 2019 was celebrated by the SMA community, and that has opened the door for more clinical trials. The current administration advisory for Zolgensma is an intravenous infusion for children under the age of two years and below a certain weight [20]. The subsequent clinical trials share the common objective of increasing the range of patients to whom Zolgensma is available. The SMART study is assessing the safety, tolerability, and efficacy of intravenous infusions of Zolgensma in patients with SMA that weigh between 18.7 lbs and 46.3 lbs [22]. The STRONG and STEER studies are investigating the safety of an intrathecal infusion of Zolgensma in patients living with SMA [23].

SMA has an incidence of one in 10,000 live births and affects the mobility of infants younger than one year of age [24]. SMA Type 1 can not only reduce a child’s ability to sit independently but, if left untreated, can lead to respiratory failure and eventually death by eight months [2]. The significance of exploring an effective form of gene therapy can provide information on what is the most optimal form of treatment for those affected with SMA Type 1. Zolgensma is currently used as an alternative therapy to treat infants diagnosed with SMA Type 1. Understanding its efficacy, side effects, and safety concerns can confirm whether it is the right medication to distribute to patients and increase their chances of mobility and survival. This review analyzed the challenges and considerations for Zolgensma and determined if they outweighed the efficacy of treatment.

After reviewing each literarture study, we noticed positive results regarding the prescribing of Zolgensma. Two studies done by Lowes and Dabbous support the use of Zolgensma as a more effective treatment for SMA Type 1. In Lowe’s study, all 12 subjects who received Zolgensma at a therapeutic dose had a greater increase in CHOP-INTEND points. Out of the 12, 11 patients by the end of the study achieved a CHOP-INTEND score greater than or equal to 40, a number that is not usually reached in patients with SMA Type 1. Similarly, Dabbous’s study compared the usage and efficacy of Zolgensma with that of Nusinersen and concluded the former drug was the most efficient. 100% of the patients who received Zolgensma increased in CHOP-INTEND points by four or more, while only 71% of those in the Nusinersen increased in their score [24]. In regard to motor function and survival rate, these studies are examples of those that have proven Zolgensma to be the more optimal drug for SMA Type 1. Another evident finding in both studies is that those who were given Zolgensma earlier in life (less than three months in particular) showed a quicker improvement in independent sitting and motor performance than later [5]. While those who received the drug improved significantly, studies showed that the “early dosing” subgroup scored higher than the “late dosing” subgroup in the CHOP-INTEND scale [5]. Exploring the dosage timeline for this therapy can help us better understand the optimal time to provide it to patients.

A safety consideration of Zolgensma that was explored was its adverse effects and whether they outweighed the overall efficacy of the drug. In particular, the hepatotoxicity that followed patients using the drug was further researched. This was added to the FDA’s safety information on Zolgensma and stated the warning as “acute serious liver injury” if taken [25]. Chand’s study showed elevated levels of alanine transaminase (ALT) and aspartate transaminase (AST) in 90% of patients one week and one month post-Zolgensma treatment. While this is a safety concern that may cause hesitancy in those prescribing the drug, it is also important to state that the elevations of AST and ALT were able to resolve and decrease by the end of the study [4]. Specifically, those whose levels were elevated in the first week decreased by the second, and those in the first month decreased by the second. In addition, the prophylactic use of prednisolone has been suggested for those taking Zolgensma to prevent a significant increase in liver enzymes post-administration [4]. With close monitoring of liver enzyme levels and the addition of prophylactic medications, this adverse reaction can be easily managed while taking Zolgensma. Practitioners should mitigate risk through appropriate monitoring and intervention [13].

**Figure 1:**
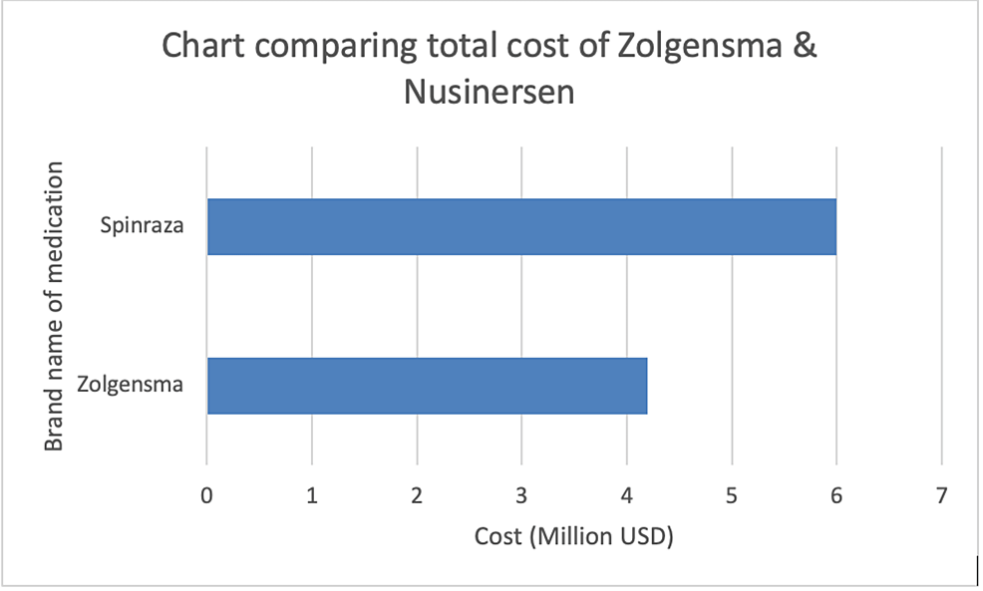
Chart comparing total cost of Zolegensma and Nursinersen

Regarding the cost of the drug, a study completed by Malone concluded that it is more cost-efficient than alternatives such as Nusinersen. A single-dose intravenous injection of Zolgensma was estimated to cost $4.2 million in a lifetime. In comparison, the cost of Nusinersen, an injection that is administered every four months, was estimated at $6.3 million in a lifetime [11] (Table 1 and Figure 1).

**Table 1:** Comparison of cost and administration between Zolgensma and Nusinersen.

Medication	Zolgensma	Nusinersen
Frequency of Dosage	Single Dose	Every 4 months
Estimated Cost per Dose	$4.2 million	$125,000
Injection Type	Intravenous	Intrathecal
Estimated Total	$4.2 million	$6 million

Critics of Zolgensma lament the cost of this therapy, but they only do so because of a short-sighted view. This therapy is labeled as the most expensive ever, but the truth is that there are scores of other treatment plans that cost more cumulatively [20]. This is a one-time infusion that replaces the need for continuous treatment at intervals. Since the patient will not be injected multiple times as with other treatment plans, there is a reduced risk of infusion site-related infection or pain [20].

While these studies showed positive outcomes with Zolgensma, some limitations were present in the selection criteria, specifically for participants with pre-existing liver damage. Of the 90% of patients with elevated liver enzymes post-Zolgensma, 61% had mild elevations before dosage [4]. It is unclear how significantly the drug contributes to the hepatotoxicity of patients, especially in those with pre-existing liver damage. Further research must be done on the adverse effects on participants both with and without pre-existing liver failure. In addition, it is important to explore safety and efficacy outcomes and observe whether they vary across social groups, such as gender and race.

Highlighted Studies

A major advantage of Zolgensma is its level of effectiveness once introduced early enough, as supported by a follow-up analysis for 12 infants with SMA Type 1 and a different analysis of 21 studies [24,12]. An Infant Test of Neuromuscular Disorders was conducted by the Children’s Hospital of Philadelphia, using the Hammersmith Infant Neurological Examination (HINE) method, and it was concluded that Zolgensma has a better survival rate than the gene-therapy treatment Nusinersen [13] (Table 2).

**Table 2:** A Novel gene therapy Onasemnogene Abeparvovec (Zolgensma) for Spinal Muscular Atrophy (SMA)

Study	Population	Amount of Doses Given	Duration of Treatment	Diagnostic Test Utilized	Outcome/Results
Day et al., 2021. Non-randomized, open label, single-group assignment [26]	22	Single	18 months	Achievement of Independent Sitting for at Least 30 Seconds, Event-Free Survival, Ability to Thrive, Ventilatory Support Independence	1/22 patients died from treatment. 2/22 patients did not complete the study, one due to withdrawal from the study and the other due to adverse events. 19/22 patients completed the study and passed the diagnostic test utilized.
Al-Zaidi et al., 2019. Non-randomized, open label, single-group assignment [12]	12	Single	1 year, with 2-year observation	Noninvasive ventilation (NIV), swallow function, speaking ability, head control, assisted sitting requirements	7/12 patients did not require NIV; 11/12 patients had stable/improved swallowing function (tested by ability to feed orally); 11/12 patients were able to speak; Zolgensma treatment decreased the need for pulmonary and nutritional support and improved the function of motor skills.
Mendell et al., 2016. Non-randomized, open label, single-group assignment [13]	15	Single injection at 2 different doses. 12 patients received 2.0x10^14 vg/kg (vector genomes) and 3 patients received 6.7x10^13 vg/kg.	2 years	Unassisted sitting time, duration of standing and walking unassisted, and CHOP INTEND	A high dose was given to 12 patients. From those 12, 11 were able to sit unassisted, nine were able to roll over, 11 could feed themselves orally and speak, and two could independently walk. And four patients had high levels of serum aminotransferase. This elevation was reversed by prednisolone.
Kevin Strauss, Francesco Muntoni et al. (2021) [14]	15	Single	Ongoing	Ventilation, feeding tube support, developmental milestones	All patients are alive, and none used ventilatory or feeding tube support at any time. Eight of the 15 patients showed remarkable progress. Two patients had achieved the primary efficacy endpoint of “stands alone within the normal developmental window”.

## Conclusions

Zolgensma, though highly criticized for its cost, is an effective one-time treatment for SMA. It is more cost-effective than comparable therapies, Evrysdi and Spinraza, and requires less time for treatment. The succinct features of Zolgensma will most likely cause this medication to become popular among physicians in the future. There is substantial evidence of improved outcomes when Zolgensma is administered early to children under two years of age. With the efficacy of this treatment option, physicians treating patients with SMA can be more confident in making an early diagnosis of SMA, which will improve the chances of survival and quality of life for their patients. Adverse events associated with this gene therapy include local injection reactions, nausea, ALT elevations, and hypersensitivity reactions.

The range of patients who have access to Zolgensma is currently limited by age and weight due to the intravenous administration route. Future clinical trials testing the intrathecal route of delivery will potentially make Zolgensma available for older and heavier patients. Extensive studies that have focused on detecting the disease early in life will continue to improve the overall efficacy of Zolgensma in the affected population. Considering all things, Zolgensma is one of the best treatments for SMA type 1 available to date.

## Figures and Tables

**Figure 2:**
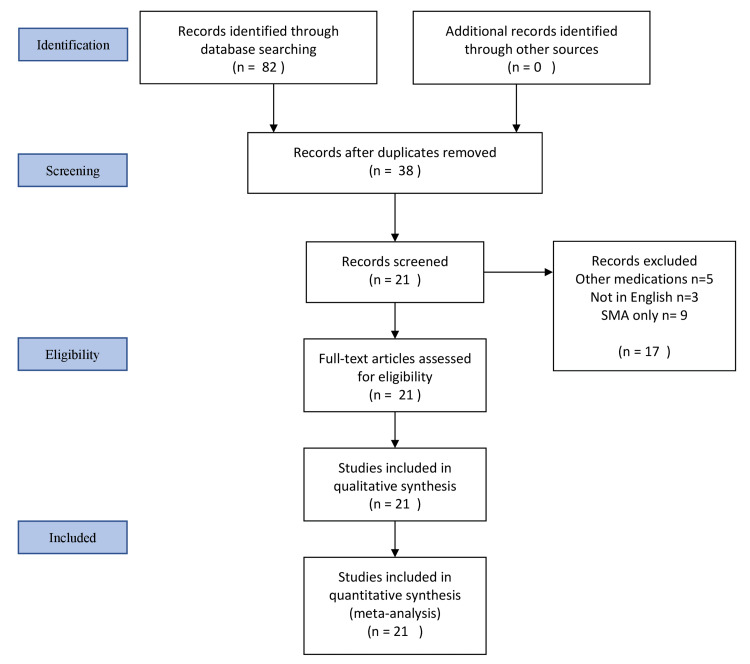
PRISMA Flow Diagram

## Appendices

PRISMA flow diagram

Figure 2 shows the PRISMA flow diagram. 
